# Dominance Between Plasmids Determines the Extent of Biofilm Formation

**DOI:** 10.3389/fmicb.2020.02070

**Published:** 2020-08-26

**Authors:** João Alves Gama, Elizabeth G. Aarag Fredheim, François Cléon, Ana Maria Reis, Rita Zilhão, Francisco Dionisio

**Affiliations:** ^1^Department of Pharmacy, Faculty of Health Sciences, UiT The Arctic University of Norway, Tromsø, Norway; ^2^Biosystems and Integrative Sciences Institute (BioISI), Faculdade de Ciências, Universidade de Lisboa, Lisbon, Portugal; ^3^Departamento de Biologia Vegetal, Faculdade de Ciências, Universidade de Lisboa, Lisbon, Portugal; ^4^Centre for Ecology, Evolution and Environmental Changes (cE3c), Faculdade de Ciências, Universidade de Lisboa, Lisbon, Portugal

**Keywords:** conjugative plasmids, biofilms, interactions, dominance, *Escherichia coli*

## Abstract

Bacterial biofilms have an impact in medical and industrial environments because they often confer protection to bacteria against harmful agents, and constitute a source from which microorganisms can disperse. Conjugative plasmids can enhance bacterial ability to form biofilms because conjugative pili act as adhesion factors. However, plasmids may interact with each other, either facilitating or inhibiting plasmid transfer. Accordingly, we asked whether effects on plasmid transfer also impacts biofilm formation. We measured biofilm formation of *Escherichia coli* cells harboring two plasmid types, or when the two plasmids were present in the same population but carried in different cells. Using eleven natural isolated conjugative plasmids, we confirmed that some indeed promote biofilm formation and, importantly, that this ability is correlated with conjugative efficiency. Further we studied the effect of plasmid pairs on biofilm formation. We observed increased biofilm formation in approximately half of the combinations when both plasmids inhabited the same cell or when the plasmids were carried in different cells. Moreover, in approximately half of the combinations, independent of the co-inhabitation conditions, one of the plasmids alone determined the extent of biofilm formation – thus having a dominant effect over the other plasmid. The molecular mechanisms responsible for these interactions were not evaluated here and future research is required to elucidate them.

## Introduction

Bacteria may live as unicellular planktonic organisms or as part of biofilms, which are complex bacterial communities embedded in a polymeric matrix. These complex structures have an important impact in medical and industrial environments because they often confer protection to bacteria against harmful agents, such as antimicrobials and host immune components, and constitute a source from which microorganisms can disperse (Costerton et al., 1999; Hall-Stoodley et al., 2004; Flemming et al., 2016). Clinically, biofilms are estimated to be involved in at least 65% of bacterial infections (Joo and Otto, 2012). Additionally, due to the close proximity between cells in biofilms, these structures can potentially favor horizontal gene transfer, mediated by mobile genetic elements such as plasmids, contributing to the spread of antibiotic resistance genes [reviewed in Molin and Tolker-Nielsen (2003), Sorensen et al. (2005), Stalder and Top (2016))]. Plasmids, concurrently, can also promote biofilm formation (Ghigo, 2001; Reisner et al., 2006).

*Escherichia coli* strains carrying plasmid F are good biofilm formers while plasmid-free isogenic strains are not (Ghigo, 2001). This effect is no longer observed when using a derivative of plasmid F not expressing sex pili, showing that these appendages can act as adhesion factors to increase biofilm formation (Ghigo, 2001). Plasmid-mediated biofilm promotion is more evident when plasmid-carrying strains invade a population of plasmid-free cells (Ghigo, 2001; Krol et al., 2013). The role of plasmids as biofilm developers was confirmed by screening 403 *E. coli* strains (Reisner et al., 2006) of which almost 50% developed better biofilms when co-cultured with plasmid-free cells than when cultured alone. This sub-collection of strains was shown to carry conjugative plasmids. Those plasmid-free cells that acquired plasmids during co-culture also became better biofilm producers.

While sex-pili promote cellular contact in early phases of biofilm formation (Ghigo, 2001), plasmids may enhance biofilm formation by other means (D’Alvise et al., 2010; Lim et al., 2010) allegedly acting in parallel or in subsequent phases. As an example, the conjugative machinery of plasmid F also stimulates *E. coli* to synthesize colanic acid and curli proteins, which play a role in biofilm maturation (May and Okabe, 2008). Other studies, focused on plasmid R1drd19, showed that the expression of several *E. coli* chromosomal genes changed due to the presence of this plasmid (Barrios et al., 2006; Yang et al., 2008). Such changes increase cell aggregation, promote quorum sensing AI-2 signaling and decrease motility, thus resulting in enhanced biofilm formation. These examples affecting the different biofilm phenotypes are due to molecular cross-talk between plasmid and chromosome, that was additionally shown to be specific of plasmid-host combinations. For instance, plasmid pKJK5 decreases *Pseudomonas putida*’s ability to form biofilms and increases that of *Kluyvera sp.* but does not affect *E. coli* (Roder et al., 2013).

Plasmids carry accessory genes that shape biofilm formation. As an illustration, plasmids belonging to the incompatibility subgroup IncX1, as plasmids pOLA52 and pMAS2027, commonly encode type 3 fimbriae (Burmolle et al., 2012), which mediate attachment to surfaces and promote biofilm formation (Burmolle et al., 2008; Ong et al., 2009). The effect of plasmid pOLA52 was to foster an increase in biofilm formation in *Salmonella enterica* serovar Typhimurium, *Kluyvera sp.* and *Enterobacter aerogenes*, whereas in *Klebsiella pneumoniae*, that also chromosomally encodes other type 3 fimbriae, the effect was lower (Burmolle et al., 2008). On the other hand, common antibiotic resistance genes frequently carried in plasmids can also affect biofilm development. Specifically, overexpression of efflux pumps leading to tetracycline resistance induces expression of surface structures that promote biofilm formation, while class A and D β-lactamases inhibit biofilm formation, possibly by preventing the correct assembly of such structures (Gallant et al., 2005; May et al., 2009). Intriguingly, conjugative pili may sometimes decrease the ability of conjugative plasmids to enhance biofilm formation (Ong et al., 2009), and even non-conjugative plasmids may enhance biofilm formation despite not expressing sex pili (Teodosio et al., 2012; Mathlouthi et al., 2018; Nakao et al., 2018).

Altogether, the role of plasmids in biofilm formation seems to be complex, not being dictated in a single direction nor by single factors, also obeying interactions with the host chromosome. Another layer to this already complex behavior is that multiple mobile genetic elements can be found in bacterial communities, even at the intracellular level, and interact among themselves. The existence of such interactions can shape individual effects, leading to a diversity of behaviors (Dionisio et al., 2019).

As far as it concerns the effect of specific and multiple plasmid interactions on biofilm formation, research is scarce. It has been shown that a conjugative and a non-conjugative plasmids interacted in a synergistic manner to promote biofilm formation (Dudley et al., 2006). Moreover, antagonistic interactions were reported when cells in a population carried different, but related, plasmids such that they expressed surface exclusion (a process by which plasmid-carrying cells become less capable to engage in conjugation with cells harboring a related plasmid) which prevented cell contacts and biofilm formation (Reisner et al., 2006).

In order to expand the limited research on this subject, we aim to evaluate how conjugative plasmids interactions affect biofilm formation. We show that the ability of conjugative plasmids to promote biofilm formation is correlated with their conjugative efficiency. Furthermore, in half of the strains with multiple plasmids analyzed, one of the plasmids exhibited a dominant effect on biofilm development.

## Materials and Methods

### Bacterial Strains, Plasmids and Media

We used the following bacterial strains: *E. coli* K12 MG1655 Δ*ara*, as plasmid-free, containing each of the 11 natural conjugative plasmids (summarized in Table 1), or containing the 33 possible combinations (due to incompatibility or selective markers) of two plasmids. For single-plasmid conjugation experiments, we used *E. coli* K12 MG1655 as the recipient strain. All these strains were produced in prior work (Gama et al., 2017). We conducted all experiments in Lysogeny Broth (LB) without antibiotics, unless otherwise stated.

**TABLE 1:** Plasmids used in this work.

Plasmid	Incompatibility Group	Resistance Markers^a^	Source^b^
R16a	IncA/C	Amp^e^, Kan	S.C.K. – C.E.N.
R57b	IncA/C	Amp^f^, Cm	S.C.K. – C.E.N.
F^c^	IncF I	Tet	I. Matic (C.N.R.S.)
R124	IncF IV	Tet	S.C.K. – C.E.N.
R1	IncF II	Amp^e^, Cm, Kan, Str	G. Koraimann (Graz Univ.)
R1drd19^d^	IncF II	Amp^e^, Cm, Kan, Str	G. Koraimann (Graz Univ.)
RN3	IncN	Str, Tet	S.C.K. – C.E.N.
R702	IncP-1	Kan, Str, Tet	S.C.K. – C.E.N.
RP4	IncP-1	Amp^e^, Kan, Tet	S.C.K. – C.E.N.
R388	IncW	Tmp	S.C.K. – C.E.N.
R6K	IncX	Amp^e^, Str	DSMZ

*^*a*^Underlined markers were used for selection of transconjugants acquiring single plasmids, while all markers were used when selecting for transconjugants carrying two plasmids. Amp, ampicillin (100 μg/mL); Cm, chloramphenicol (30 μg/mL); Kan, kanamycin (100 μg/mL); Str, streptomycin (100 μg/mL); Tet, tetracycline (20 μg/mL); Tmp, trimethoprim (100 μg/mL). ^*b*^C.N.R.S, Centre National de la Recherche Scientifique; DSMZ, Leibniz-Institut DSMZ German Collection of Microorganisms and Cell Cultures; S.C.K.,C.E.N. – Belgian Nuclear Research Centre; Graz Univ., Karl-Franzens-Universität Graz, Institute of Molecular Biosciences. ^*c*^Constitutive for conjugation (Yoshioka et al., 1987; Fernandez-Lopez et al., 2016). ^*d*^De-repressed natural mutant of R1 (Koraimann et al., 1991). ^*e*^Class A (TEM) β-lactamase (Matthew and Hedges, 1976; Szabo et al., 2016). ^*f*^class D (OXA) β-lactamase (Matthew and Hedges, 1976).*

### Growth Rate Measurements

We cultivated the plasmid-free strain, 11 single-plasmid carrying strains and six strains carrying two plasmids (combinations of F, R16a, R388 and R6K) in LB overnight at 37°C with agitation. We diluted these cultures 100-fold in LB, and added 250 μL (∼2 × 10^7^ CFU/mL) to a 96-well plate. We conducted the experiments with three biological replicates, each consisting of three technical replicates. We incubated the plates overnight at 37°C in an EPOCH 2 microplate reader (BioTek Instruments, Inc) with continuous shaking, taking OD_600_ measurements every 10 min. We used GrowthRates v3.0 (Hall et al., 2014) to estimate growth rates. We calculated growth rates for each biological replicate as the average of the technical replicates (after discarding those with a *R*^2^ correlation coefficient <0.95). Finally, we estimated the growth rate of each strain relative to the plasmid-free strain. Thus, we calculated the ratio between the growth rate of each biological replicate of each strain by the mean growth rate of the plasmid-free strain.

To estimate the population size of cultures for each of the strains mentioned above, we plated serial 10-fold dilutions of the respective overnight cultures in LB agar plates and incubated them overnight at 37°C, to estimate the CFU/mL. We performed this experiment with three biological replicates.

### Biofilm Formation Assays

We measured biofilm formation in four conditions: (i) the plasmid-free strain cultivated alone (reference values); (ii) single-plasmid carrying strains co-cultivated with the plasmid-free strain; (iii) double-plasmid carrying strains co-cultivated with the plasmid-free strain; (iv) two different single-plasmid carrying strains co-cultivated together.

To assay biofilm formation, we followed the protocol by Christensen et al. (1985). Briefly, we cultivated the strains in 96-well plates (U-bottom, polystyrene, Greiner bio-one) containing 200 μL of LB per well. After overnight incubation at 37°C with agitation, we mixed 5 μL of the donor and 5 μL of the recipient strain in 190 μL of LB in a new 96-well plate. We then transferred 5 μL of each mix (∼2.5 × 10^5^ CFU per strain) into 195 μL of LB in a new plate, which we incubated for 24 h at 37°C without agitation, inside plastic bags to prevent evaporation.

### Biofilm Formation Quantification

We washed the plates three times to remove unattached cells and then stained them with crystal violet (0.1%) for 15 min [adapted from Christensen et al. (1985)]. We dissolved the crystal violet with ethanol (96%) and measured absorbance at 595 nm (Microplate Reader Tecan Spec 11.A Rainbow). We collected the values for the monoculture of the plasmid-free strain, for each plate and averaged them after removing outliers (see section “Statistics”). We normalized all well-measurements of the same plate by dividing each value by the average of the monoculture of the plasmid-free strain.

**FIGURE 1:**
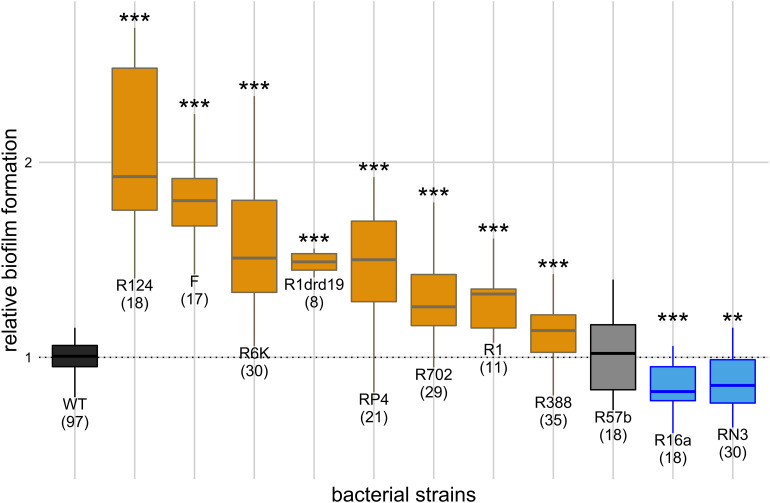
Effect of single plasmids on biofilm formation. *Y* axis represents biofilm formation relative to the plasmid-free strain (WT). Annotations under the boxes indicate the identity of the plasmids and, in parenthesis, the number of biological replicates. Results of Dunnett’s multiple comparison test against the plasmid-free strain are indicated above the boxes as: **p-value < 0.01; ***p-value < 0.001. Box color indicates the phenotype qualitatively, such that biofilm formation: increased significantly (orange), decreased significantly (blue), or did not change significantly (light gray). Dark gray indicates the plasmid-free reference strain (WT).

We performed at least six biological replicates for each experiment. As the number of replicates varies for different strains/conditions, we indicate the number of biological replicates used (after removal of outliers) in Figures 1, 3 and 4.

### Conjugation Efficiency

To measure the conjugative efficiency of the 11 individual plasmids, we set up 96-well plates as described above for biofilm assays, with two differences: (1) we co-cultivated each *E. coli* K12 MG1655 Δ*ara* carrying a single plasmid with plasmid-free *E. coli* K12 MG1655 (*ara*^+^), and (2) we incubated the plates for 2 h. After incubation, we diluted a 100 μL culture sample in 0.9% NaCl and plated serial dilutions on tetrazolium arabinose (TA) agar to quantify donor and recipient CFU/mL (red and white colonies, respectively) and on M9 minimal media supplemented with one antibiotic (Table 1) selective for the specific plasmid to quantify transconjugants. We incubated TA plates overnight and M9 plates for 48 h, at 37°C. We estimated conjugation efficiency as$\frac{T}{\sqrt{D\times R}}$, where T, D, and R are the CFU/mL of transconjugants, donors and recipients, respectively. We performed three biological replicates for each experiment.

When co-cultivating two different single-plasmid carrying strains together, we repeated the biofilm assay to quantify the proportion of cells that carried two plasmids at the end of the experiment. After the 24 h incubation, we diluted a 100 μL culture sample in 0.9% NaCl and plated appropriate dilutions on LB agar to quantify total cells and on LB agar supplemented with all antibiotics selective for the two plasmids (Table 1). We incubated the plates overnight at 37°C. We performed this experiment for the six combinations of strains carrying plasmids F, R16a, R388, and R6K, with three biological replicates.

### Homologues of Type 3 Fimbriae

We used the BLASTX algorithm to search in the nucleotide sequence of plasmid R6K^1^ for homology with the type 3 fimbriae of *K. pneumoniae* (accession no. M55912), considering an e-value < 10^–5^.

### Statistics

We performed statistical tests in R version 3.5.1, available at http://www.rstudio.com/ (R Core Team, 2018).

We verified normality of the biofilm formation values obtained for each sample (Shapiro-Wilk test) after removing outliers (no values outside [*Q*_1_ − 1.5 × IQR, *Q*_3_ + 1.5 × IQR], where Q1 and Q3 are, respectively, the first and third quartiles and IQR is the interquartile range (_*Q*3–*Q*1_).

We estimated linear regression and correlations between biofilm formation and conjugation efficiency, growth rate and plasmid size, after excluding outliers according to the studentized residuals method (Venables and Ripley, 2002).

We conducted one-way ANOVAs controlled for heteroscedasticity, and when significant we further performed Tukey’s or Dunnett’s multiple comparison tests. Non-paired Welch *t*-tests were used to compare two samples.

## Results

### The Effect of Single Plasmids on Biofilm Formation

We measured how co-cultivation of the plasmid-free strain with each of the 11 single-plasmid carrying strains affected biofilm formation relatively to being cultivated alone. The effect of the 11 natural conjugative plasmids, belonging to six different incompatibility groups, on biofilm formation is shown in Figure 1. Raw OD_595_ reads for negative controls (uninoculated LB) varied from 0.05 to 0.07, while reads for inoculated experimental assays varied from 0.10 to 0.51 with raw reads for the plasmid-free strain varying from 0.10 to 0.35. Compared with the plasmid-free strain (one-way ANOVA, d.f. = 11, *p*-value = 1.49 × 10^–39^, followed by Dunnett’s multiple comparison test), eight plasmids increased the strain’s ability to form biofilms, two decreased it and one did not have a significant effect. Cells carrying plasmid R124 exhibited the highest biofilm formation, while those carrying plasmid R16a or RN3 exhibited less biofilm formation than the plasmid-free strain.

**FIGURE 2:**
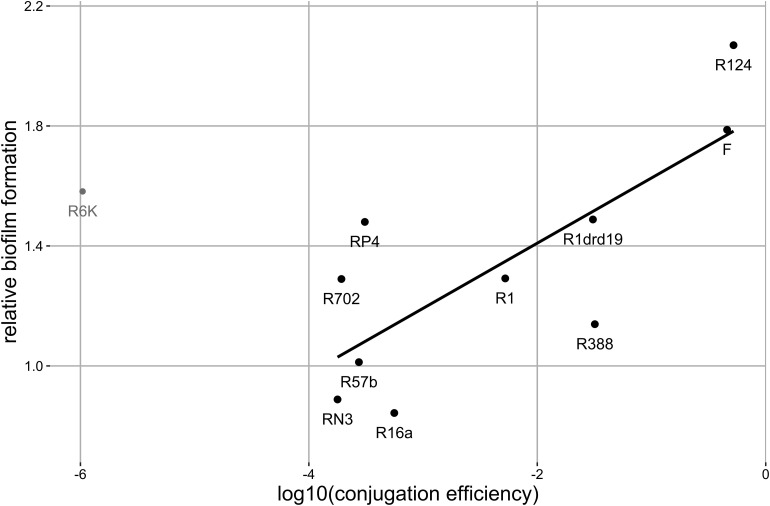
Correlation between biofilm formation and conjugation efficiency. Each data point represents the mean value for each plasmid (number of replicates as in Figure 1). The black line represents the regression line (*y* = 0.22x + 1.84, *R*^2^ = 0.59, σ^2^ = 0.07) for black points, where y is the biofilm production relative to the plasmid-free strain and *x* is the log10 of the conjugation rate. Gray point represents the excluded outlier. Significant correlation: Pearson method, two-sided, d.f. = 8, ρ = 0.77, *p*-value = 0.009.

Since plasmids can enhance biofilm formation via conjugative pili, we checked for a correlation between the ability to form biofilms and conjugation efficiency. This correlation was significant (Pearson method, two-sided, d.f. = 8, *p*-value = 0.009, ρ = 0.77) after outlier (plasmid R6K) exclusion (Figure 2). The correlation was no longer significant if R6K was included (Pearson method, two-sided, d.f. = 9, *p*-value = 0.16, ρ = 0.45). The result shows that, in general, a high conjugation rate correlates with an increased ability to form biofilms.

Plasmid R6K stood as an outlier in the above correlation, increasing biofilm formation despite its low conjugative efficiency. Plasmids belonging to the incompatibility subgroup IncX1 commonly encode type 3 fimbriae, presumably acquired from *K. pneumoniae*, which enhances biofilm formation (Burmolle et al., 2012). Plasmid R6K is related to these plasmids since it belongs to the incompatibility subgroup IncX2. Therefore, we searched for genes coding for type 3 fimbriae in the sequence of R6K. Regardless, we did not identify any homologues.

We also investigated whether growth rate changes due to plasmid carriage affected biofilm formation. Compared to the plasmid-free strain (one-way ANOVA, d.f. = 11, *p*-value = 0.00015, followed by Dunnett’s multiple comparison test), four of the eleven plasmids affected host growth rates significantly (Supplementary Table S1). Strains carrying plasmids R57b, R124, R388, and R6K displayed significantly reduced growth rates, with effects ranging from 12.44 to 26.44%. Despite the difference in growth rates, all strains attained overnight population sizes (i.e., carrying capacity) of ∼2 × 10^9^ cells/mL. The correlation between biofilm formation and relative growth rates was not significant neither with inclusion (Pearson method, two sided, d.f. = 9, *p*-value = 0.74, ρ = −0.11) nor exclusion of the two outliers F and R124 (Pearson method, two sided, d.f. = 7, *p*-value = 0.41, ρ = −0.32). It has been hypothesized that increasing plasmid sizes could be associated with higher fitness costs (Zünd and Lebek, 1980; Cheah et al., 1987; Vogwill and MacLean, 2015), however plasmid sizes could as well increase the probability of carriage of genes promoting biofilm formation. We observed no significant correlation (Pearson method, two sided, d.f. = 8, *p*-value = 0.053, ρ = 0.62) between relative growth rates and plasmid size (Supplementary Table S1). Likewise, the correlation between biofilm formation and plasmid size was not significant (Pearson method, two sided, d.f. = 8, *p*-value = 0.89, ρ = 0.05), even after excluding outliers (Pearson method, two sided, d.f. = 5, p-value = 0.33, ρ = 0.43).

### Intra and Intercellular Plasmid Interactions

**FIGURE 3:**
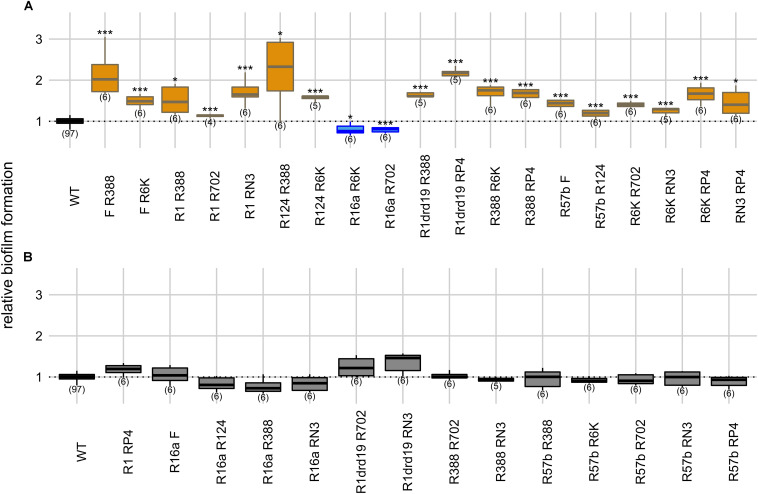
Intracellular effects of two plasmids on biofilm formation. Panels indicate significant **(A)** and non-significant effects **(B)** on biofilm formation relatively to the plasmid-free strain. *Y* axis represents biofilm formation relative to the plasmid-free strain (WT). Plasmid combinations are indicated in the *x* axis and the number of biological replicates below the boxes (in parenthesis). Results of Dunnett’s multiple comparison test against the plasmid-free strain are indicated as: *p-value < 0.05; **p-value < 0.01; ***p-value < 0.001. Box color, as in Figure 1, indicates the phenotype qualitatively, such that biofilm formation: increased significantly (orange), decreased significantly (blue), or did not change significantly (light gray in panel **B**). Dark gray indicates the plasmid-free reference strain (WT).

We measured biofilm formation of 33 strains carrying different combinations of two plasmids (from the 11 plasmids) to understand the effect of plasmids’ intracellular interactions on this phenotype. In this setup, the plasmid-free strain was co-cultivated with each of the 33 double-plasmid carrying strains. When compared with the plasmid-free strain alone (one-way ANOVA, d.f. = 33, *p*-value = 1.55 × 10^–31^, Dunnett’s multiple comparison test), 17 of the strains carrying two plasmids displayed increased biofilm formation and two strains displayed decreased biofilm formation (Figure 3). R16a, one of the two plasmids that, alone, decreases biofilm formation, was present in the two combinations exhibiting decreased biofilm formation.

We measured growth rates of the strains carrying six combinations of two plasmids (F, R16a, R388, and R6K) relatively to the plasmid-free strain (Supplementary Table S1). Four of these strains displayed significantly different growth rates (one-way ANOVA, d.f. = 6, *p*-value = 0.007, Dunnett’s multiple comparison test), but all six attained population sizes of ∼2 × 10^9^ cells/mL as described for plasmid-free and single-plasmid carrying strains. The combinations F/R388, F/R6K, R16a/R6K, and R16a/R388 reduced host’s growth rate significantly but affected biofilm formation differently. Among the two combinations not significantly affecting growth rates, R388/R6K increased biofilm formation, while F/16a did not affect it significantly. Therefore, the relative growth rate of the strains does not seem to be a good predictor of the effect on biofilm formation, supporting the previous results of no significant correlation between the two variables.

**FIGURE 4:**
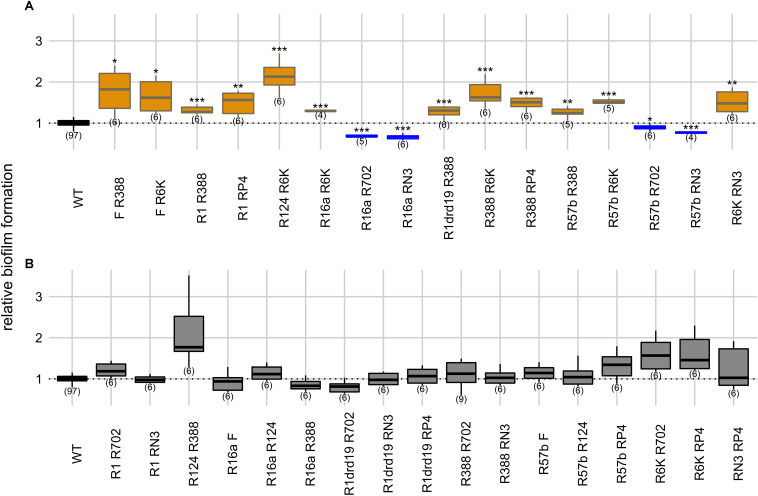
Intercellular effects of two plasmids on biofilm formation. Panels indicate significant **(A)** and non-significant effects **(B)** on biofilm formation relatively to the plasmid-free strain. *Y* axis represents biofilm formation relative to the plasmid-free strain (WT). Plasmid combinations are indicated in the *x* axis and the number of biological replicates below the boxes (in parenthesis). Results of Dunnett’s multiple comparison test against the plasmid-free strain are indicated as: *p-value < 0.05; **p-value < 0.01; ***p-value < 0.001. Box color, as in Figures 1 and 3, indicates the phenotype qualitatively, such that biofilm formation: increased significantly (orange), decreased significantly (blue), or did not change significantly (light gray in panel **B**). Dark gray indicates the plasmid-free reference strain.

Plasmids can be present simultaneously in the same bacterial population but carried in different host cells. Therefore, we also measured the ability conferred by two plasmids to form biofilms when carried in two different strains. In this setup two single-plasmid carrying strains were co-cultivated. When compared with the plasmid-free strain alone (one-way ANOVA, d.f. = 33, *p*-value = 1.76 × 10^–45^, Dunnett’s multiple comparison test), the ability to form biofilms increased in 12 cases, and decreased in four (Figure 4). R16a and RN3, the two plasmids that, alone, decrease biofilm formation, are involved in three of the four cases.

In this “intercellular” experimental condition, cells harboring one of the plasmids serve both as donors and as recipients of plasmids carried by the other cells. This should result in more cellular interactions since both cells can express sex pili and can promote more stable mating pairs during the conjugative process (Dionisio et al., 2019). However, we observed no statistical difference (Fisher’s exact test, two-sided, *p*-value = 0.39) between intra- and intercellular conditions regarding the number of cases of increase, decrease or no effect on biofilm formation. One potentially confounding factor is that a high proportion of the populations may end up carrying both plasmids in the “intercellular” experiment. Therefore, we measured the proportion of cells carrying both plasmids for the six combinations of the four plasmids F, R16a, R388, and R6K. After 24 h of incubation, the populations where plasmid F (one of the plasmids with the highest conjugative efficiency) was present, 30–59% of cells carried both plasmids, while the populations with the other three plasmid combinations displayed 9–17% of cells carrying both plasmids (Supplementary Table S2). In comparison, the “intracellular” experiment started with 50% of the population already harboring both plasmids. Therefore, the impact of cells with both plasmids on biofilm formation appears to be low.

We then assessed the effect of the two conditions combination-wise, by comparing (Welch’s *t*-test) intracellular versus intercellular effects for each combination of plasmids (Supplementary Table S3). In 17 of the 33 combinations, biofilm formation did not differ significantly between conditions (Welch’s *t*-test, two-sided, *p*-values > 0.05). In nine combinations, biofilm formation was greater in intracellular conditions, while in the remaining seven combinations biofilm formation was greater in intercellular conditions. Overall, these results suggest that biofilm formation is not consistently favored by one of the two types of cellular interactions and depends on specific plasmid combinations.

### One of the Plasmids Frequently Exerts a Dominant Effect on Biofilm Formation

In the previous section we showed that the effect of multiple plasmids on biofilm formation tends to be combination-specific, and therefore depends on how the plasmids interact. We checked whether one of the plasmids has a determinant effect (over the other) on biofilm production. For that, we simultaneously compared biofilm formation of the population carrying two plasmids (either one strain carrying two plasmids – intracellular condition –, or two strains each carrying one plasmid – intercellular condition) and each of the two strains carrying a single plasmid (one-way ANOVA followed by Tukey’s multiple comparison test). The amount of biofilm (B) produced is represented as B_AB_ for the population carrying two plasmids and as B_A_ and B_B_, respectively, for each of the strains carrying a single plasmid, A or B. The analysis can produce several outcomes:

(I) Dominance – when: B_AB_ ≈ B_A_ AND B_AB_ ≠ B_B_
(II) Increased – when: B_AB_ > B_A_ AND B_AB_ > B_B_
(III) Decreased – when: B_AB_ < B_A_ AND B_AB_ < B_B_
(IV) Intermediate – when: B_B_ < B_AB_ < B_A_, OR when: B_AB_ ≈ B_A_ AND B_AB_ ≈ B_B_ AND B_A_ ≠ B_B_
(V) Undetermined – when: B_AB_ ≈ B_A_ AND B_AB_ ≈ B_B_ AND B_A_ ≈ B_B_

Where ≈ indicates non-significant differences and, ≠ indicates significant differences. It should be noted that there are two possible cases of Dominance: the biofilm produced by the strain carrying two plasmids does not differ from that produced by the single plasmid-carrying strain that produced more biofilm (Dominance by the Highest) or less biofilm (Dominance by the Lowest).

The results are summarized in Supplementary Table S4. Dominance prevailed in both intracellular and intercellular conditions, representing more than half (36/66 = 54.5%) of the outcomes: 16 cases by the Highest and 20 cases by the Lowest. Three cases exhibited Increase (all in intracellular conditions) and seven cases exhibited Decrease. There are 13 Intermediate cases and a minority of cases (7/66) were categorized as Undetermined. In these undetermined cases, we observed no significant difference (Fisher’s exact test, two-sided, *p*-value = 0.34), when comparing intracellular and intercellular conditions in terms of the six possible outcomes.

## Discussion

Conjugative plasmids have been shown to enhance bacterial biofilm formation due to the expression of conjugative pili (Ghigo, 2001; Reisner et al., 2006). Here we confirmed that most of the tested conjugative plasmids enhanced biofilm formation (Figure 1) and further showed that this ability is correlated with conjugative efficiency (Figure 2) which supports previous studies (Ghigo, 2001; Reisner et al., 2006). Nevertheless, we observed that plasmid R6K is an exception to this rule since it displays low conjugative efficiency but increased biofilm formation. This indicates and is in line with other findings (Gallant et al., 2005; Barrios et al., 2006; Burmolle et al., 2008; May and Okabe, 2008; Yang et al., 2008; May et al., 2009; Ong et al., 2009; D’Alvise et al., 2010; Lim et al., 2010; Nakao et al., 2018) showing that besides sex pili, other factors like adhesion factors or altered gene expression due to plasmid-chromosome cross-talk affect biofilm formation. Examples of these include IncX1 plasmids that encode type 3 fimbriae promoting biofilm formation (Burmolle et al., 2012). However, although the IncX2 plasmid R6K is related to IncX1 plasmids, we did not find sequences encoding such an appendage.

Not all plasmids led to increased biofilm formation (Figure 1), and two of them (RN3 and R16a) even decreased it. In fact, lower biofilm formation due to plasmids was reported before (Roder et al., 2013) and several factors, like the action of β-lactamases (Gallant et al., 2005; May et al., 2009), can influence it. Since R16a, one of the five plasmids encoding β-lactamases, was the only one leading to lower biofilm formation, we infer that coding for a β-lactamase is not a sufficient feature to diminish the development of biofilms. Plasmid RN3, on the other hand, does not encode such enzymes. Altogether, other factors could be accountable for this phenomenon, such as quorum sensing inhibition, alteration of cell-surface, or downregulation of chemotaxis [reviewed in Rendueles and Ghigo (2012)].

Plasmid carriage is known to affect host growth rate (Carroll and Wong, 2018; Gama et al., 2018) and could have consequences on biofilm formation. The method used in this work can only detect substantial growth effects. To precisely measure growth effects a more robust method would be preferred (Morton et al., 2014). Yet, although we detected significant growth rate reduction associated with four plasmids, it was not significantly correlated with their effect on biofilm formation.

Cross-talk between plasmids and chromosomes affects biofilm formation (Barrios et al., 2006; May and Okabe, 2008; Yang et al., 2008; May et al., 2009; Lim et al., 2010; Roder et al., 2013; Nakao et al., 2018), and interactions between plasmids can also impact biofilm development (Dudley et al., 2006). We previously tested intracellular and intercellular interactions in 33 pairs of conjugative plasmids and showed that conjugative efficiency tends to decrease when both plasmids were present in the same cell, and to increase if they were carried in different cells (Gama et al., 2017). Here we tried to relate how plasmid interactions may affect biofilm formation. In essence, we showed that, although biofilm formation differs between these two co-inhabitation conditions (intra and intercellular), it does not seem to be consistently favored by one or the other (Figures 3, 4 and Supplementary Table S3). Rather, biofilm formation is determined by particular combinations of plasmids.

Dominance was the main outcome in both intra- and intercellular conditions (Supplementary Table S4), meaning that one of the two plasmids determines the extent of biofilm formation. The features responsible for the effects observed were not evaluated here. We consider that they could be multiple. For instance, plasmid R16a had a dominant effect in 5/6 of intracellular interactions (the other one was undetermined), and could thus encode a single factor that consistently decreases biofilm formation, exerting a dominant effect when another plasmid inhabits the same cell. On the other hand, plasmid R388 when alone had a weak effect on biofilm formation and in 9/10 of intracellular interactions was recessive, but in combination with R702 decreased biofilm formation. These two plasmids inhibit each others’ conjugative transfer (Olsen and Shipley, 1975; Fong and Stanisich, 1989; Getino et al., 2017), which could explain reduced biofilm development. However, interactions between other plasmid genes or even more complex plasmid-chromosome-plasmid cross-talk cannot be ruled out. Future research is required to dissect the mechanisms responsible for the plasmid interactions affecting biofilm formation.

A weakness of this work is the usage of lab strains, known to be weak biofilm formers and we report small albeit significant effects. To further expand on the relevance of our findings beyond lab strains, future studies comprising clinical strains carrying multiple plasmids would be of relevance to address the importance of plasmid interactions on biofilms of representative *E. coli* isolates circulating in the community.

Our work focused on plasmid interactions during the early phases of biofilm formation on polystyrene plates. Nevertheless, studies with different conditions and environments are important to assess the robustness of our results. It is relevant to explore how bacterial growth phases and different biofilm stages affect plasmid interactions. For some plasmids longer incubation times are required to promote biofilm formation (Ghigo, 2001), and, while sex pili are important for initial adhesion, other plasmid factors affect later biofilm maturation (May and Okabe, 2008). Initial adhesion is also determined by the type of surface (e.g., polystyrene, glass or cultured cells) (Dudley et al., 2006), and the rate of plasmid transfer also varies depending on the physical region of the biofilm (Krol et al., 2011). Therefore, plasmid interactions may depend on the habitat and culture conditions and contribute to distinct adherence patterns. In conclusion, not only the mechanisms behind plasmid interactions, but also the interplay between these interactions and the environment should be the target of future research.

## Data Availability Statement

All datasets generated for this study are included in the article/Supplementary Material.

## Author Contributions

JG, RZ, and FD conceived the study. JG, AR, and EF designed the experiments. JG, EF, and FC performed the experiments. JG and FD analyzed the data. JG wrote the first draft of the manuscript, with contributions from AR, RZ, and FD. All authors contributed to manuscript revision, read and approved the submitted version.

## Conflict of Interest

The authors declare that the research was conducted in the absence of any commercial or financial relationships that could be construed as a potential conflict of interest.

## Footnotes

1 ftp://ftp.sanger.ac.uk/pub/pathogens/Plasmids/R6K.dbs
